# 547 Volume-based Feeds: A Quality Improvement Project for Better Nutrition

**DOI:** 10.1093/jbcr/irad045.144

**Published:** 2023-05-15

**Authors:** Emma Levine, Elizabeth Dale Slater, Hanna Slutsky, Olatundun Ladele, Stephen Gondek, Anne Wagner

**Affiliations:** Vanderbilt University, Nashville, Tennessee; Vanderbilt University Medical Center, Nashville, Tennessee; Vanderbilt University Medical Center, Nashville, Tennessee; Meharry Medical College, Nashville, Tennessee; Vanderbilt University Medical Center, Nashville, Tennessee; Vanderbilt University Medical Center, Nashville, Tennessee; Vanderbilt University, Nashville, Tennessee; Vanderbilt University Medical Center, Nashville, Tennessee; Vanderbilt University Medical Center, Nashville, Tennessee; Meharry Medical College, Nashville, Tennessee; Vanderbilt University Medical Center, Nashville, Tennessee; Vanderbilt University Medical Center, Nashville, Tennessee; Vanderbilt University, Nashville, Tennessee; Vanderbilt University Medical Center, Nashville, Tennessee; Vanderbilt University Medical Center, Nashville, Tennessee; Meharry Medical College, Nashville, Tennessee; Vanderbilt University Medical Center, Nashville, Tennessee; Vanderbilt University Medical Center, Nashville, Tennessee; Vanderbilt University, Nashville, Tennessee; Vanderbilt University Medical Center, Nashville, Tennessee; Vanderbilt University Medical Center, Nashville, Tennessee; Meharry Medical College, Nashville, Tennessee; Vanderbilt University Medical Center, Nashville, Tennessee; Vanderbilt University Medical Center, Nashville, Tennessee; Vanderbilt University, Nashville, Tennessee; Vanderbilt University Medical Center, Nashville, Tennessee; Vanderbilt University Medical Center, Nashville, Tennessee; Meharry Medical College, Nashville, Tennessee; Vanderbilt University Medical Center, Nashville, Tennessee; Vanderbilt University Medical Center, Nashville, Tennessee; Vanderbilt University, Nashville, Tennessee; Vanderbilt University Medical Center, Nashville, Tennessee; Vanderbilt University Medical Center, Nashville, Tennessee; Meharry Medical College, Nashville, Tennessee; Vanderbilt University Medical Center, Nashville, Tennessee; Vanderbilt University Medical Center, Nashville, Tennessee

## Abstract

**Introduction:**

Nutrition is a critical component to recovery in burn patients. Their heightened metabolic demand is well established. In our burn population, patients often received significantly less volume of feeds than they were prescribed, primarily due to stoppages for sedations and procedures. We, therefore, developed a quality improvement (QI) project to help improve the quantity of feeds received.

**Methods:**

Under the direction of nursing QI leadership and our burn nutritionist, we established an outcome measure of increasing average feeds received from baseline 68% of those prescribed to greater than 75% of those prescribed. Using a process of iterative changes, we initially developed a calculator for volume-based feeds to make up the volume lost during times tube feeds were paused. Nursing staff gave feedback, and we ultimately used a chart for adjusted volumes based on the total 24 hour goal and number of hours remaining in which to make up the goal. On a bi-weekly basis, we examined the amount of tube feeds as a percent of total prescribed prior to and after the interventions.

**Results:**

Early results have demonstrated improvements in the percent of tube feeds a burn patient is receiving. Prior to the interventions patients were receiving about 68% of the prescribed tube feeds and within 6 weeks of project initiation, patients were receiving 82% of the prescribed tube feeds (Figure 1). In addition, variability in feeds received decreased markedly, as have number of days above the target.

**Conclusions:**

In conclusion, this has been an effective QI intervention as we surpassed our initial target within 6 weeks of initiation. Next steps are to consider increasing the target to 90% of prescribed feeds, as well as investigating adequacy of feeds using nitrogen balance in select patients. Future directions for this project include assessing outcome measures such as graft take and hospital length of stay.

**Applicability of Research to Practice:**

Purpose is to share our methods in quality improvement to aid others efforts facing similar challenges in the care of the critically ill burn patient.

